# Combination Erlotinib-Cisplatin and Atg3-Mediated Autophagy in Erlotinib Resistant Lung Cancer

**DOI:** 10.1371/journal.pone.0048532

**Published:** 2012-10-31

**Authors:** Jasmine G. Lee, Reen Wu

**Affiliations:** Department of Internal Medicine, Division of Respiratory Medicine, University of California Davis, Davis, California, United States of America; H. Lee Moffitt Cancer Center & Research Institute, United States of America

## Abstract

Tyrosine kinase inhibitors such as erlotinib are commonly used as a therapeutic agent against cancer due to its relatively low side-effect profile and, at times, greater efficacy. However, erlotinib resistance (ER) in non-small cell lung cancer is being recognized as a major problem. Therefore, understanding the mechanism behind ER and developing effective regimens are needed. Autophagy’s role in cancer has been controversial and remains unclear. In this study, we examined the effectiveness of low dose erlotinib-cisplatin combination in erlotinib resistant lung adenocarcinoma (ERPC9) cells and the role of autophagy in ER. ERPC9 cells were established from erlotinib sensitive PC9 cells. Appropriate treatments were done over two days and cell survival was quantified with Alamar Blue assay. LC3II and regulatory proteins of autophagy were measured by western blot. Small interfering RNA (siRNA) was utilized to inhibit translation of the protein of interest. In ERPC9 cells, combination treatment induced synergistic cell death and a significant decrease in autophagy. At baseline, ERPC9 cells had a significantly higher LC3II and lower p-mTOR levels compared to PC9 cells. The addition of rapamycin increased resistance and 3-methyladenine sensitized ERPC9 cells, indicating autophagy may be acting as a protective mechanism. Further examination revealed that ERPC9 cells harbored high baseline Atg3 levels. The high basal Atg3 was targeted and significantly lowered with combination treatment. siRNA transfection of Atg3 resulted in the reversal of ER; 42.0% more cells died in erlotinib-alone treatment with transfection compared to non-transfected ERPC9 cells. We reveal a novel role for Atg3 in the promotion of ER as the inhibition of Atg3 translation was able to result in the re-sensitization of ERPC9 cells to erlotinib-alone treatment. Also, we demonstrate that combination erlotinib-cisplatin is an effective treatment against erlotinib resistant cancer by targeting (down-regulating) Atg3 mediated autophagy and induction of apoptotic cell death.

## Introduction

Lung cancer remains the leading cause of cancer-related deaths and has one of the lowest survival rates among all cancers, with a reported five-year survival of 13% [1]. Lung cancer can be broadly categorized into two main groups for prognostic and treatment purposes: small cell lung cancer (SCLC) and non-small cell lung cancer (NSCLC). Of all lung cancers, 85% are NSCLC [2] and is further subdivide into three groups based on their histological characteristics: squamous cell carcinoma, large cell carcinoma, and adenocarcinoma [2]–[4]. NSCLC tends to be less sensitive to chemotherapy than SCLC, and even with surgical resection of early stage primary tumors, up to 50% of patients show recurrence of their primary cancers [4], [5]. Because of this, effective chemotherapy regimens are needed and at times, systemic chemotherapy is the only option for locally advanced tumors and/or metastatic disease.

Platinum based chemotherapeutic agents such as *Cis*-diamminedichloroplatinum (II) (cisplatin) have been traditionally the main agents used to treat NSCLC and have been shown to improve patient survival [6]. However, cisplatin carries significant toxic side-effects, especially at high doses, such as: nausea, emesis, renal failure, ototoxicity and neurotoxicity due to its non-specific cytotoxic effects on both cancer and normal cells [7], [8].

More recently, there is an ongoing interest in research, development, and production of anti-cancer agents that target specific pathological pathways, allowing such agents to have less toxic profiles and lower risk for side effects. One such Food and Drug Administration (FDA) approved agent used in lung adenocarcinoma is erlotinib. Erlotinib is a tyrosine kinase inhibitor (TKI) that inhibits the epidermal growth factor receptor (EGFR) through competitive inhibition of the ATP-binding site in the tyrosine kinase domain [9], and subsequently reducing downstream proliferative signaling pathways [10]. EGFR is overly expressed in many cancers, including 40–80% of NSCLC [11], [12]. Mutations associated with EGFR, like mutations leading to the overexpression of EGFR, are considered critical mechanism for tumorigenesis as EGFR is involved in many regulatory growth processes including proliferation, apoptosis, adhesion, invasion, and migration [2], [10], [13]. Erlotinib has been shown to be an effective treatment for NSCLC harboring EGFR mutations [14]. However, about 10 to 14 months after starting treatment, some lung cancers develop erlotinib resistance (ER) leading to recurrence [15], [16]. There have been few studies on the mechanisms and involved pathways of acquired resistance to TKIs, but this topic still remains controversial and relatively unclear warranting further elucidation of the possible molecular mechanisms of resistance [17].

Autophagy has been classically understood to be a mechanism for cell protein homeostasis and degradation of injured cellular components and/or organelles [18], [19]. Its role is critical as impaired autophagy is linked with various human diseases including cancer [19], [20]. Autophagy has also been described to act as a “second” pathway of programmed cell death (the other being apoptosis) and conversely a protective mechanism for cell integrity [20], [21]. However, its role in cancer and cancer resistance remains unclear: does autophagy act as a mechanism to promote cancer resistance or promote cancer cell death [22], [23]? Therefore, determining and understanding the role of autophagy in ER is important.

In order to improve the treatment of NSCLC, it is essential to understand the molecular mechanism underlying ER and find regimens that are effective in treating erlotinib resistant cancers. Thus, in this study, we examined the effectiveness of low-dose erlotinib-cisplatin combination treatment in erlotinib resistant lung adenocarcinoma and determine the role of autophagy in ER. We also identify a key regulatory protein in the autophagy pathway that is able to modulate ER.

## Materials and Methods

### Cell Lines and Cell Culture

An erlotinib sensitive PC9 cell line, a human lung adenocarcinoma with overexpression of EGFR, was kindly gifted from Dr. Halmos (Columbia University) [24]. It was maintained in RPMI 1640 media containing 10% fetal bovine serum (FBS) with Antibiotic-Antimycotic (Invitrogen, Carlsbad, CA). The ERPC9 cell line was established by culturing PC9 cells in 5% FBS culture media containing erlotinib. Cells were initially maintained at an erlotinib concentration of 33 nM (IC50) and the dose was gradually increased over a period of 12 weeks until the final concentration of erlotinib was 10 µM. Then, through the use of single-cell cloning techniques in which only actively dividing cells were chosen (indicating resistance), ERPC9 cells were established. Then, ERPC9 cells were maintained in 10% FBS in RPMI 1640 containing the final established erlotinib concentration of 10 µM.

**Figure 1:**
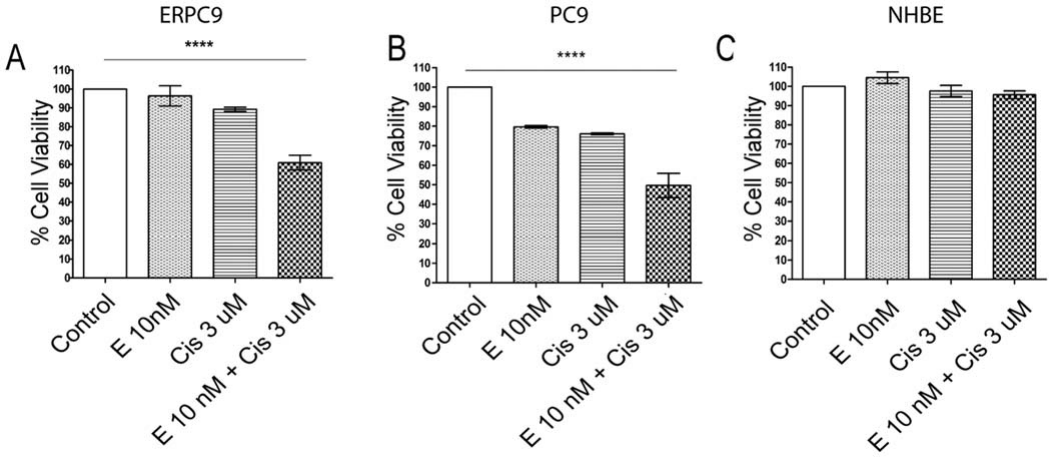
Two day treatment of PC9, erlotinib resistant PC9 cells and normal human bronchial epithelial cells. * Cell viability was quantified using Alamar Blue Assay. The control group acted as the standard for cell viability and cell death. (A) In ERPC9 cells, combination treatment induced significantly more cell death than single drug treatments (p<0.0001). Erlotinib-alone resulted in 96.6% survival, cisplatin 89.3%, and combination 61.1%; this effect was synergistic (CI of 0.12). (B) In the parental PC9 cells, combination treatment induced significantly more cell death than single drug treatments (p<0.0001) erlotinib-alone resulted in 79.6% survival, cisplatin 76.1%, and combination 49.6%; this effect was synergistic (CI of 0.45). (C) In NHBE cells, erlotinib alone, cisplatin alone, and combination treatment demonstrated minimal cell death or change in viability among the four groups (p = 0.958) indicating minimal toxicity with combination treatment.

Normal human bronchial epithelial tissues were obtained from the National Disease Research Interchange (Philadelphia, PA) [25]–[27]. Tissues were not collected from patients diagnosed with lung-related diseases. Protease-dissociated bronchial epithelial cells were plated on transwell chambers (Corning; 24 mm) at 5×10^4^ cells/cm^2^ in bronchial epithelial growth medium (Lonza). After 4–7 days in an immersed culture condition or when cultures reached confluency, cells were transferred to an air-liquid interface (ALI) culture condition in a Ham’s F12/DMEM (1∶1) with the addition of the following eight factors: transferrin (5 µg/ml), insulin (4 µg/ml), cholera toxin (20 ng/ml), epidermal growth factor (10 ng/ml), dexamethasone (0.1 µM), bovine hypothalamus extract (15 µg/ml), BSA (0.5 mg/ml), and all-*trans*-retinoic acid (30 nM), which facilitated polarization and mucociliary differentiation. NHBE cells were cultured for 7 days after transferring to ALI. All cells were maintained at 37°C in a humidified incubator with 5% CO2.

**Figure 2:**
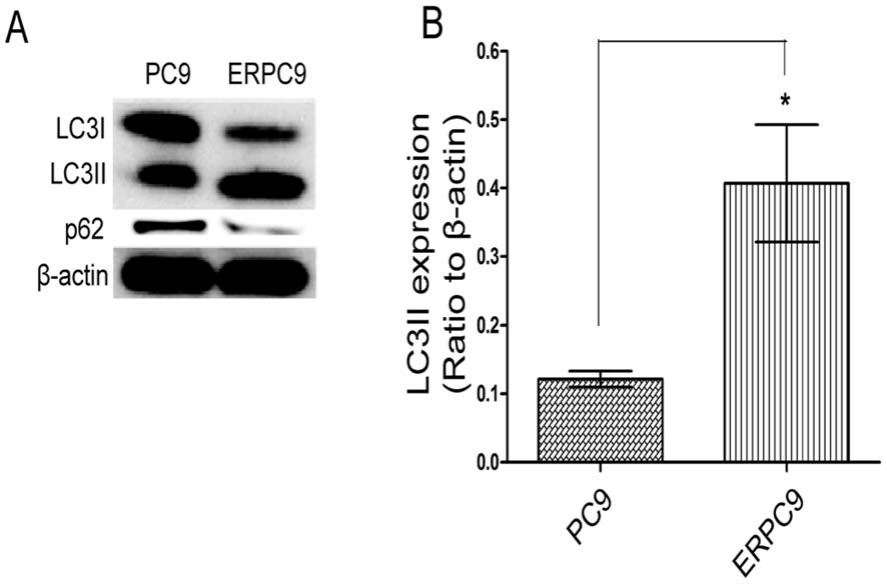
LC3II and LC3I levels in PC9 cells and erlotinib resistant PC9 cells. * (A) Autophagy levels were assessed by western blot for LC3II and p62. (B) ERPC9 cells had significantly higher levels of LC3II than in PC9 cells (p = 0.030) and significantly lower levels of p62 than in PC9 cells (p<0.0001); indicating higher baseline autophagy levels in erlotinib resistance cells. Concordantly, it can be seen that there were significantly lower levels of LC3I in ERPC9 cells compared to PC9 cells (p = 0.008).

### Drugs: IC50 Values and Treatment Groups

Cisplatin, rapamycin and 3-methyladenine (3-MA) were purchased from Sigma–Aldrich (St. Louis, MO, USA), and erlotinib was purchased from Selleck Chemicals (Huston, TX, USA). 3-MA was dissolved in water and all other drugs were dissolved in dimethyl sulfoxide (DMSO) to form stock concentrations of cisplatin 10 mM, erlotinib 10 µM, rapamycin 27.4 mM, and 3-MA 100 mM. 3-MA was prepared fresh each time and other drugs were maintained at −20°C and all were diluted to appropriate concentration before use. The length of treatment for all experiments consisted of two days. Cells were first plated and cultured with 10% FBS in RPMI 1640 media for 24 hours before initiation of treatment to allow cells to attach to the plate. The next day, the media was replaced with 0.1% FBS in RPMI 1640 containing appropriate drug concentrations for two days. In the determination of IC values, 96-well plates were used and dose responses were performed through the use of sensitive PC9 cells by treating cells in a serial range of drug concentrations for each drug (erlotinib or cisplatin). Official treatment groups consisted of a 2×2 factorial experimental design [28]: control (0.1% DMSO), erlotinib (10 nM), cisplatin (3 µM), and combination of erlotinib and cisplatin (10 nM+3 µM). Official drug treatments were performed in 6-well plates. Both PC9 and ERPC9 cell lines underwent drug treatments. Experiments requiring the use of rapamycin and 3-MA underwent the same protocol.

**Figure 3:**
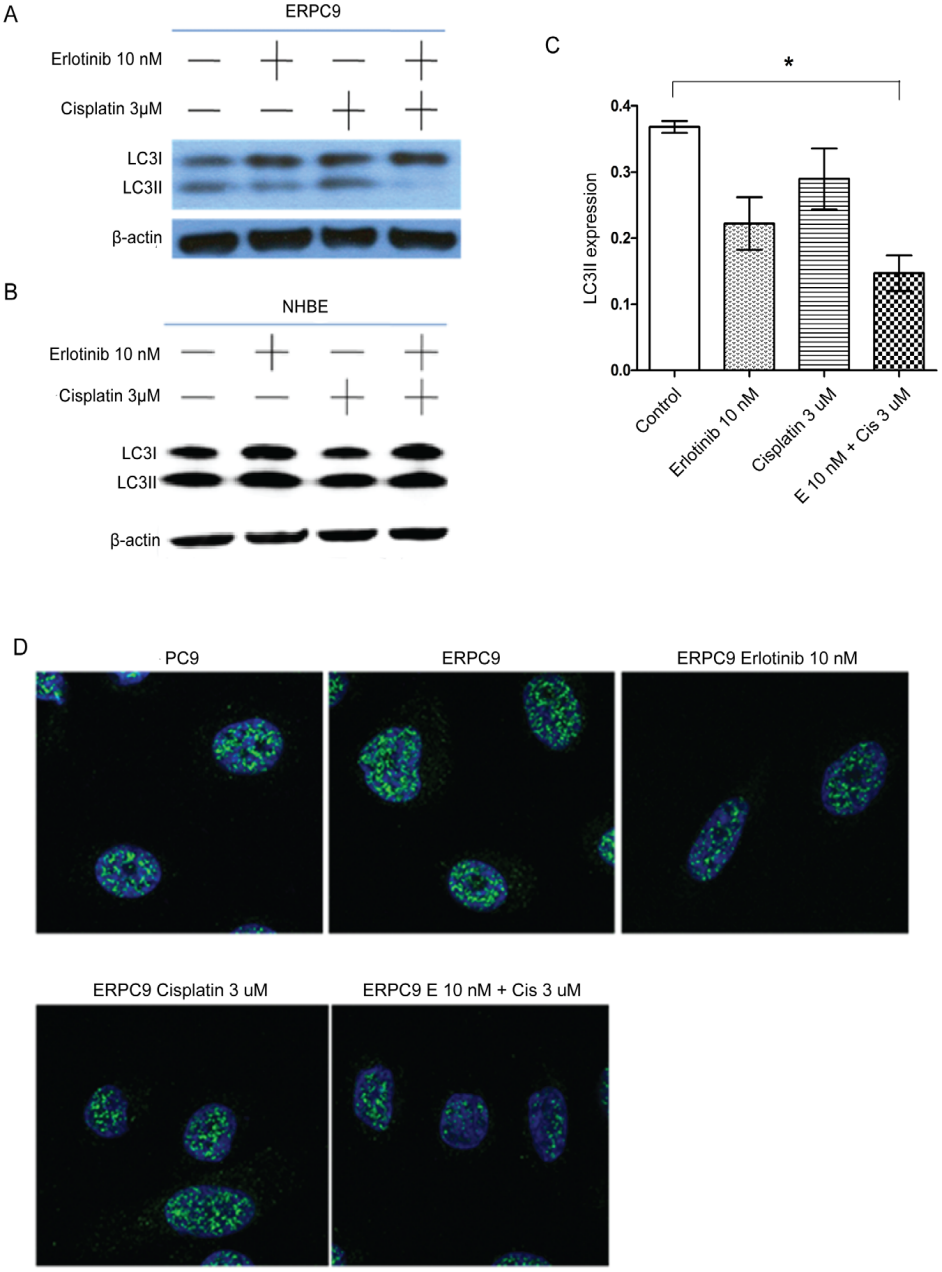
LC3II in erlotinib resistant PC9 cells and normal human bronchial epithelial cells after two-day treatment. * Autophagy levels were measured through western blot for LC3II. (A&C) In ERPC9 cells, there was a significant decrease in LC3II with combination treatment compared to other groups (p = 0.011). The decrease in LC3II was synergistic with combination treatment, mirroring the same synergistic trend for cell death. (B) There were no significant differences in LC3II levels in NHBE cells (p = 0.958). (D) Significantly greater LC3II (green) fluorescence was observed in ERPC9 cells compare to PC9 cells (p<0.0001). In ERPC9 cells that underwent combination treatment, a significant decrease in green fluorescence was seen compared to all other groups (p<0.0001). Green = LC3 and Blue = DAPI.

### Cell Viability Assay

Cell viability was quantified using the Alamar Blue Assay (Invitrogen, Carlsbad, CA, USA). Upon the completion of drug treatment, the original media was replaced with media containing 10% Alamar Blue dye and incubated for 1 hr in a 37°C humidified incubator with 5% CO2. Cell viability and death were then measured at 530 nm excitation wavelength and 590 nm emission using Packard fluorocount. The ratio of cell viability was calculated with the equation as follows: (absorbance of the treatment group)/(absorbance of the control group)×100.

**Figure 4:**
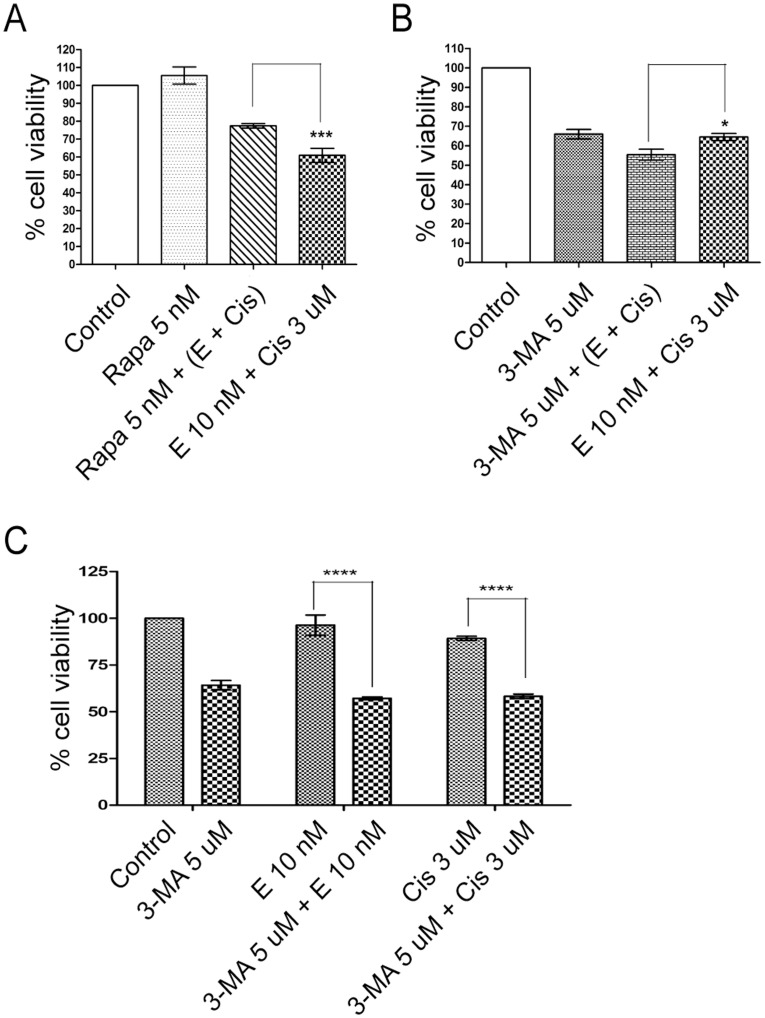
The effects of inducing and inhibiting autophagy in erlotinib resistant PC9 cells with drug treatment. * Rapamycin and 3-MA were used to assess the role of autophagy and its association with erlotinib resistance. Appropriate groups were treated with either (A) 10 nM of rapamycin or (B) 5 µM of 3-MA with combination drug treatment. In ERPC9 cells, cell survival increased 16.5% when rapamycin was added to combination treatment (p = 0.004). Compared to 3-MA treated ERPC9 cells, there was a 10.5% decrease in cell survival in combination treatment group and 9.1% decrease in 3-MA with combination treatment (p = 0.032 and p = 0.037, respectively). (C) When 3-MA was added to erlotinib-alone (p<0.0001) and cisplatin-alone (p<0.0001) treatment there was a 39.2% and 31.0% greater cell death, respectively, compared to treatment with single drug treatment alone.

### Western Blot Analysis

Cells were lysed in ice-cold RIPA buffer (Millipore, Billerica, MA) containing a combination of protease inhibitor and phosphatase inhibitor cocktails (Sigma-Aldrich). Lysated proteins were centrifuged for 10 min at 14,000 rpm at 4°C, and supernatants were quantified for protein content using Bio-Rad DC Protein Assay Kit (Bio-Rad, Hercules, CA) and a spectrophotometer measured at 650 nM. Proteins were then separated by a 4∼20% gradient SDS/PAGE gel (Thermo fisher scientific, Newington, NH) and transferred to polyvinylidene fluoride (PVDF) membranes (Bio-Rad). The membranes were blocked with 5% non-fat milk in Tris-buffered saline (TBS) containing 0.05% Tween20 (TBST) for 1 hour at room temperature (RT), and subsequently probed with anti-p62 (1∶1000, Millipore), -LC3, -Beclin 1, -P-Akt, -Akt, p-mTOR, -mTOR, -Atg3, -Atg7, -Atg5-12 complex (1∶1000, Cell signaling, Boston, MA), un-cleaved and -cleaved Caspase 3, un-cleaved and –cleaved PARP (1∶500, Cell signaling), in 2.5% non-fat milk in TBST overnight at 4°C. Then, membranes were washed 30 minutes (x3) with TBST and was probed with horseradish peroxidase-conjugated secondary antibody (1∶2000, Cell signaling) in 2.5% non-fat milk in TBST for 1 hour at RT. In order to determine equal loading and standardize the amount of protein loaded, blots were then stripped and re-blotted with monoclonal mouse anti-β-actin (Sigma-Aldrich) and the ratio of the band intensity (size×density) of interest with its associated β-actin were taken. Bands were imaged using Fuji 4000 software or via film.

**Figure 5:**
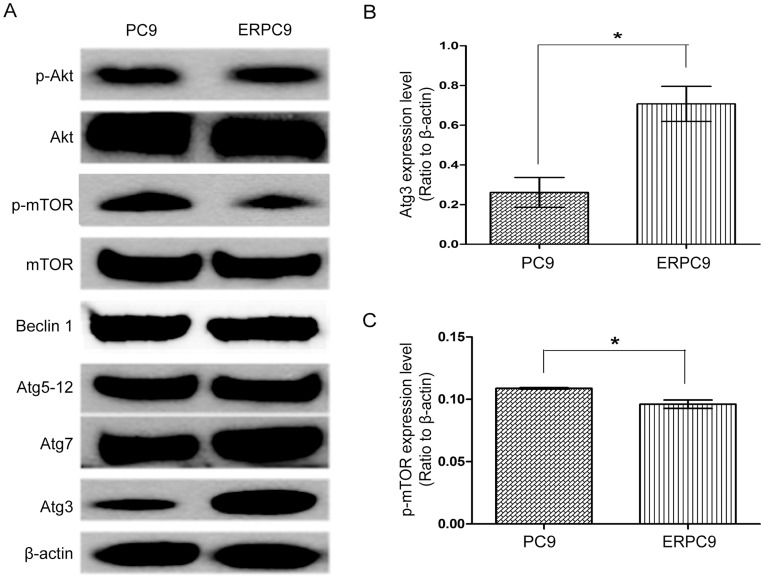
Baseline Atg3 and p-mTOR levels in erlotinib resistance PC9 cells and sensitive PC9 cells. * (A) Western blot of p-Akt, Akt, p-mTOR, mTOR, Beclin-1, Atg5-12, Atg7, and Atg3 are shown. (B) There was significantly higher Atg3 levels in ERPC9 cells compare to PC9 cells (p = 0.018) and (C) significantly lower p-mTOR level (p = 0.021) further validating that autophagy levels are higher in ERPC9 cells.

### Knock Down of Atg3 by siRNA Transfection

Small interfering RNA (siRNA) specifically targeting human Atg3 was obtained from Santa Cruz Biotechnology, Inc (Santa Cruz, CA, USA). ERPC9 and PC9 cells were plated in 12 well plates (6×10^4^ cells per well) and transfected with Atg3 siRNA for 24 hours in antibiotic free growth media; using siRNA Lipofectamine RNAiMAX and OPTI-MEM I-reduced serum medium (Invitrogen). Procedures were carried out following manufacturer’s protocol.

**Figure 6:**
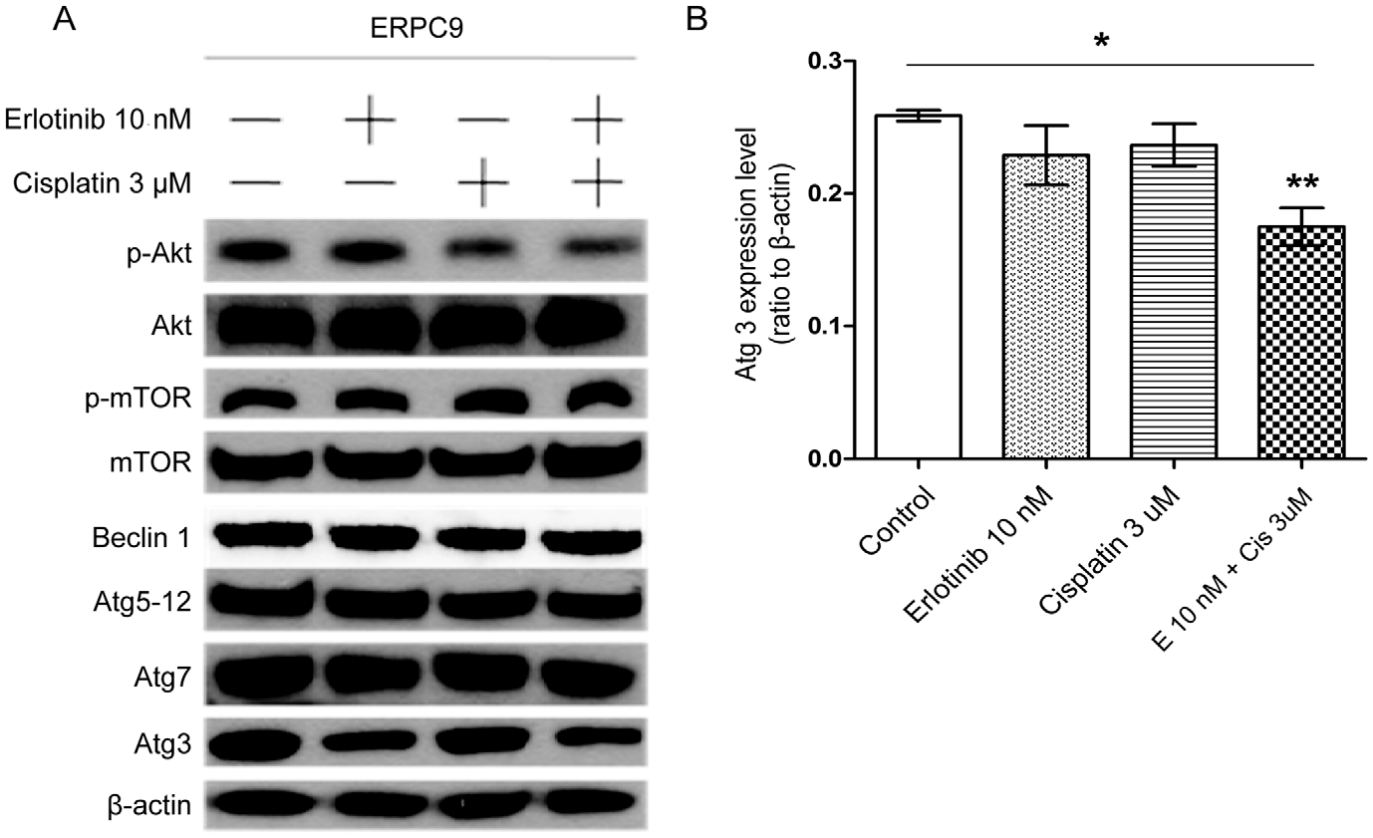
The effects of combination drug treatment on regulatory proteins of the autophagy pathway. * (A) p-Akt, Akt, p-mTOR, mTOR, Beclin 1, Atg5-12, Atg7, and Atg3 were measured through western blot analysis. (B) With erlotinib-cisplatin combination treatment, there was a significant change only in Atg3; Atg3 level was significantly decreased compared to all other groups (p = 0.028). This suggests that combination treatment may target the baseline overexpression of Atg3 in ERPC9 cells.

### Immunocytochemistry

Cells were washed with PBS twice, and fixed with 4% paraformaldehyde in PBS for 15 minutes at RT. Cells were then rewashed with PBS twice, incubated in PBS containing 0.2% Trinol X for 5 mins at RT, and blocked with blocking buffer (PBS with 3% BSA) for 5 minutes at RT. Cells were incubated with primary antibody, anti-LC3 (1∶200, Cell signaling) overnight in a 4°C dark room, washed 3 times the next day, and incubated with goat anti-rabbit IgG Alexa Flour 488 antibody (1∶1000, Invitrogen) for 1 hour at RT in a dark room. Cells were then washed with PBS and 4',6-diamidino-2-phenylindole (Dapi, Vector laboratories, Burlingame, CA) was added to stain the nuclei. Zeiss LSM700 confocal (Carl Zeiss, Germany) was used to capture images, and all images were taken at the same setting to allow consistency. Image analysis was done in a blinded fashion.

**Figure 7:**
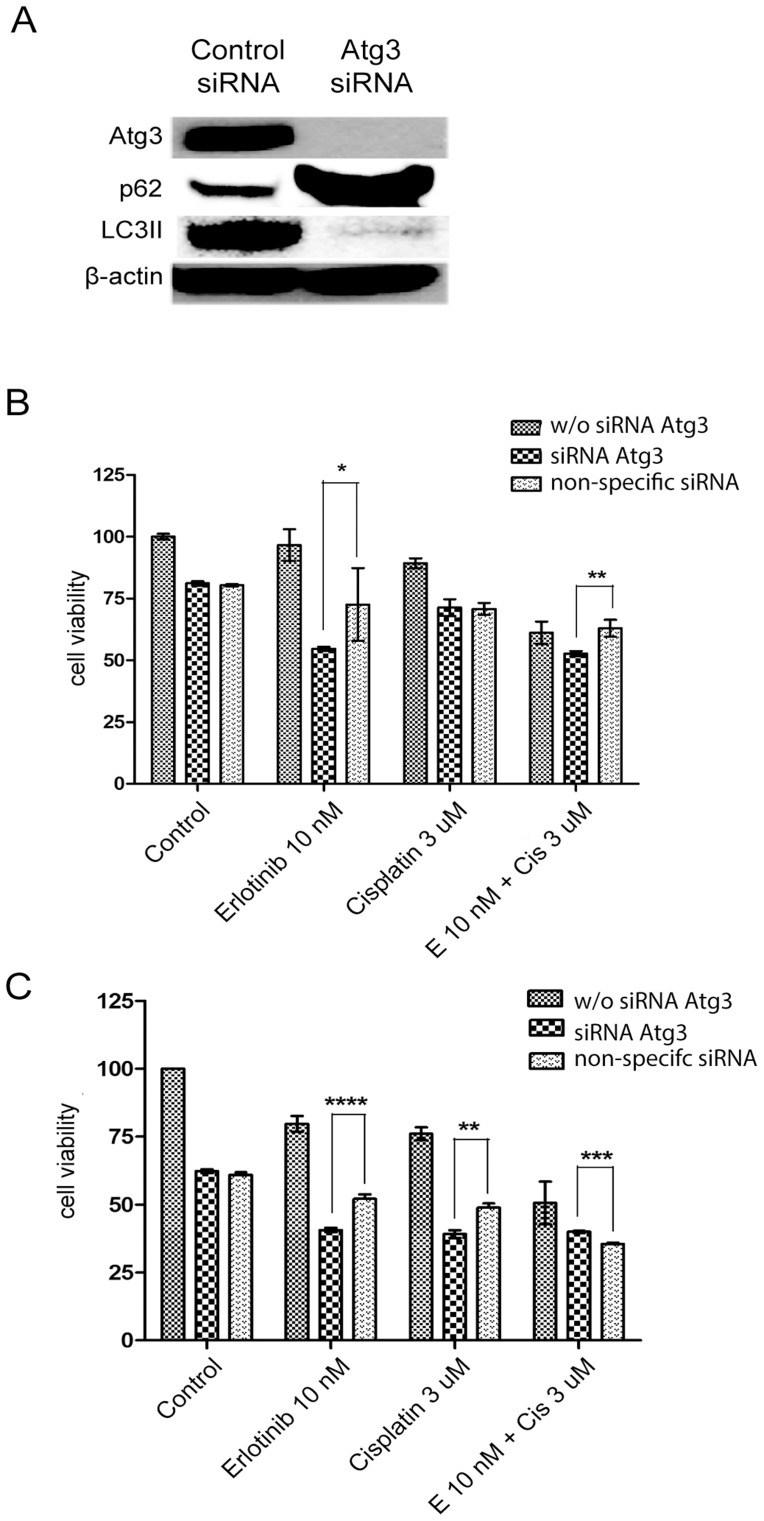
Blocking Atg3 translation reverses erlotinib resistance. * (A) Blocking Atg3 translation and autophagy with Atg3 siRNA transfection in ERPC9 cells was confirmed by western blot analysis. (B) After ERPC9 cells were transfected with Atg3 siRNA, cells became significantly more sensitive to erlotinib (p = 0.043) and combination (p = 0.002) drug treatments compared to ERPC9 cells with non-specific siRNA transfection. There was no significant difference in the cisplatin treated samples, however, (p = 0.924). (C) Atg3 siRNA transfection was able to produce a significant increase in sensitivity towards single drug treatments in the parental PC9 cell line as well.

### Pharmacologic and Statistical Analysis

Statistical analysis and determination of significant differences between groups were performed by employing two-tailed student’s t-test and analysis of variance (ANOVA) when appropriate. All statistical analysis was performed through the use of prism software (La Jolla, CA). A p-value of less than 0.05 was considered significant in this study. All experiments were performed at least three times independently.

**Figure 8:**
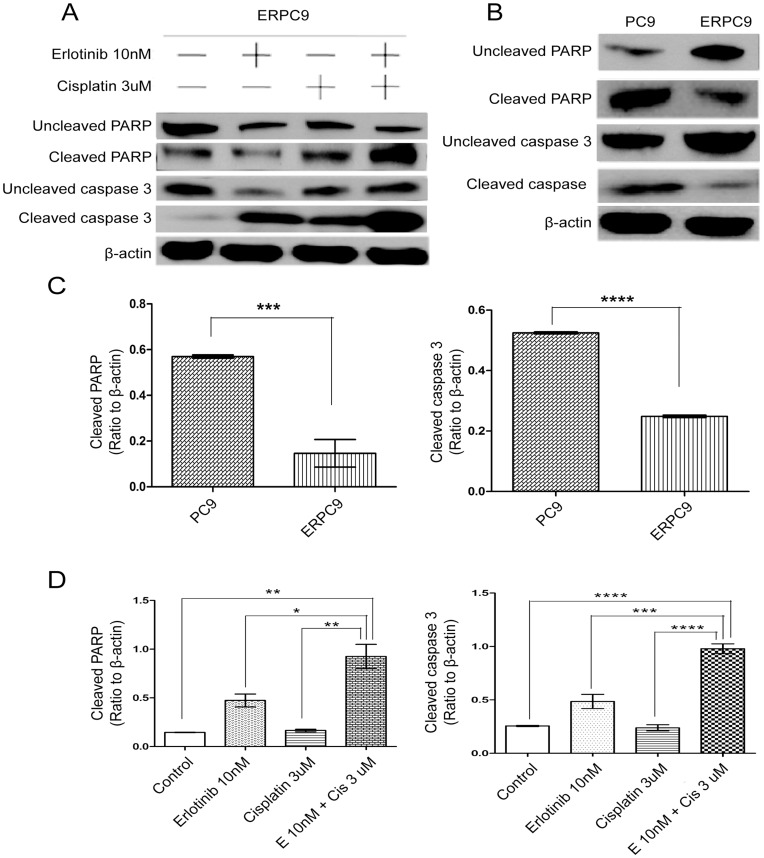
Apoptosis levels at baseline and with combination treatment. * Apoptosis was measured through western blot for cleaved caspase 3 and cleaved PARP. (A&D) In ERPC9 cells treated with erlotinib-cisplatin combination, a significant increase in cleaved caspase 3 and cleaved PARP were observed compared to non-treated cells (p = 0.045 and p = 0.007, respectively). In addition, there were significant increase in cleaved caspase 3 and cleaved PARP compared to all other groups (p = 0.030 and p = 0.003, respectively). (B&C) At baseline, there were significant lower levels in both cleaved caspase 3 and cleaved PARP in ERPC9 compared to PC9 cells (p<0.0001 and p = 0.0004, respectively).

In addition, Chou-Talalay’s method [29] was utilized to obtain the combination index (CI) to evaluate for synergism, additive effects or antagonism of combination treatment of erlotinib and cisplatin (very strong synergism, strong synergism, moderate synergism, slight synergism, additive, and antagonism were defined as CI <0.3, 0.3< CI <0.7, 0.7< CI <0.85, 0.85< CI <1, CI = 1, and CI >1, respectively).

## Results

### IC50 Values in PC9 and ERPC9 Cells

The inhibitory concentration (IC) 50 values of erlotinib and cisplatin were first established in PC9 cells before examining the effects of combining the two agents as a combination treatment in erlotinib resistant PC9 (ERPC9) cells. The determined IC50 for erlotinib was 33 nM in PC9 cells and 878 nM in ERPC9 cells (about 26 times higher than in PC9 cells) (data not shown); this confirms that ERPC9 cells were established in a manner that allowed it to become resistant and possibly mimic what is seen clinically. The cisplatin IC50 was 18 µM in PC9 cells and 40 µM in ERPC9 cells (data not shown). Given the cisplatin IC 50 did not increase significantly, this further supports that ERPC9 cells demonstrated specific resistance to erlotinib.

### Erlotinib-cisplatin Combination Treatment in ERPC9, PC9, and Normal Human Bronchial Epithelial Cells

Figure 1A and Figure 1B show the percentage of ERPC9 and PC9 cell survival after undergoing two-day drug treatments. When cells were treated with erlotinib-cisplatin combination, 61.1% of ERPC9 cells survived; this was significantly lower compared to cells treated with erlotinib-alone (96.6%) and cisplatin-alone (89.3%). In other words, combination treatment killed a significantly greater number of cells (38.9%) compared to erlotinib-alone (0.4%, p = 0.001), cisplatin-alone (10.7%, p = 0.0002) and control non-treated cells (0.0%, p<0.0001). In addition, combination treatment induced strong synergistic cell death despite ERPC9 cells’ resistance to erlotinib (CI = 0.12 as calculated through Chou-Talalay’s method [29]). A similar trend of synergistic cell death was observed in the parental PC9 cells with combination erlotinib-cisplatin treatment; cell survival after erlotinib-alone treatment was 79.6%, cisplatin was 76.1%, and combination was 49.6% (p<0.0001) (CI = 0.45). These findings suggest that the addition of cisplatin to erlotinib may be an effective regimen in killing erlotinib-resistant lung cancer cells even at low doses when used in combination.

Normal human bronchial epithelial (NHBE) cells were used to evaluate the toxicity of erlotinib-cisplatin combination treatment. Two-day treatment with erlotinib-alone, cisplatin-alone, and erlotinib-cisplatin combination demonstrated minimal cell death and no significant differences in cell death and viability among the four groups (p = 0.958), indicating a safe therapeutic index at the current dosing for non-cancer cells (Figure 1C).

### Baseline Autophagy Levels in ERPC9 Cells

Autophagy’s role in cancer has been controversial and has not been studied in great detail [30]. To examine autophagy’s role in ER, a comparison of baseline levels of autophagy between ERPC9 and PC9 cells were measured through western blot analysis for LC3II [31] and p62. As shown in Figure 2, LC3II levels were significantly higher in ERPC9 cells compared to PC9 cells (p = 0.030). In addition, p62 levels were significantly lower in ERPC9 cells than PC9 cells (p<0.0001). Both of these results suggest that ERPC9 cells harbor higher baseline autophagy levels than erlotinib sensitive PC9 cells.

### Combination Erlotinib-cisplatin Treatment and LC3II

Further examination of combination treatment in ERPC9 cells revealed significantly lower levels of LC3II when compared to all other treatment groups as seen in Figure 3A and 3C (p = 0.011). The decrease in LC3II levels on western blot analysis was synergistic, mirroring the same synergistic trend seen in cell death with combination treatment. This suggests that autophagy may be an associated mechanism of ER, and erlotinib-cisplatin combination treatment may work by targeting autophagy. Interestingly, however, there were no significant changes in LC3II in the NHBE cells (Figure 3B).

The same changes in LC3II were also visualized through immunofluorescence (Figure 3D). There was a significant decrease in LC3II fluorescence in erlotinib-cisplatin combination treated cells compared to the other treatment groups (p<0.0001).

### Modulation of Autophagy Alters Sensitivity to Treatment

In order to determine whether autophagy is an essential survival mechanism underlying ER, autophagy inducing agent, rapamycin, and inhibiting agent, 3-MA, were used. If autophagy’s role is to promote resistance and survival against treatment, it is expected that there will be increased cell survival with induction of autophagy and a converse decrease in cell survival when autophagy is inhibited after undergoing combination treatment. As hypothesized, as shown in Figure 4A, rapamycin with erlotinib-cisplatin combination treatment showed significantly greater cell survival and viability (16.5%) compared to cells that received erlotinib-cisplatin combination without rapamycin (p = 0.0004). Conversely, when 3-MA was added with erlotinib-cisplatin combination, cell survival was significantly lower (9.1%) compared to cells treated only with erlotinib-cisplatin combination treatment alone (p = 0.037) (Figure 4B). In addition, combination of 3-MA, erlotinib and cisplatin was able to kill significantly more cells than 3-MA alone (44.6% and 34.1% cell death, respectively) (p = 0.032). As Figure 4B has shown that inhibiting autophagy with 3-MA was able to sensitize the cells to combination treatment, Figure 4C shows the results of 3-MA treated ERPC9 cells with erlotinib-alone and cisplatin-alone. When 3-MA was added to erlotinib-alone (p<0.0001) and cisplatin-alone (p<0.0001) treatment there was a 39.2% and 31.0% greater cell death, respectively, compared to treatment with single drug treatment without 3-MA. These associated findings further support that autophagy may be a protective mechanism allowing greater survival in ERPC9 cells.

### Atg3 and p-mTOR in ERPC9 Cells

Figure 5 shows the results from western blot analysis of key regulatory proteins in the autophagy pathway: p-Akt, Akt, p-mTOR, mTOR, Beclin-1, Atg5-12 complex, Atg7 and Atg3. There were no significant differences in p-Akt, Akt, mTOR, Beclin-1, Atg5-12 complex, and Atg7 between ERPC9 and PC9 cells (Figure 5A). However, at baseline, p-mTOR was significantly lower in ERPC9 cells compared to PC9 cells (p = 0.021) (Figure 5C). In addition, there was a significantly higher baseline level of Atg3 in ERPC9 cells compared to PC9 cells (p = 0.018) (Figure 5B). One of the main roles of Atg3 is to convert LC3I to LC3II to promote autophagy [32] and accordingly there was a significantly lower level of LC3I in ERPC9 cells than PC9 cells (more LC3I was converted) (p = 0.008) (Figure 2A). The lower level in p-mTOR further supports the finding of higher basal levels of autophagy in ERPC9 cells. While, the extraordinary high levels of Atg3 may represent a critical component in conferring and contributing to the higher basal levels of autophagy and promotion of ER.

### Combination Treatment Targets Atg3

In order to further evaluate how combination treatment down-regulates autophagy specifically in ERPC9 cells, western blot analysis for changes in regulatory autophagy proteins was performed. As shown in Figure 6A, there were no significant changes in p-Akt, Akt, mTOR, p-mTOR, Beclin-1, Atg5-12 complex, and Atg7 among the treatment groups. There was, however, a significantly lower level of Atg3 when treated with erlotinib-cisplatin combination compared to the baseline control level (p = 0.005) (Figure 6B). This finding may suggest that erlotinib-cisplatin combination treatment may somehow target the over-expression or translation of Atg3 seen in ERPC9 cells. In addition, these findings support that Atg3 is a critical component of conferring ER and survival.

### siRNA Inhibition of Atg3 Reverses Erlotinib Resistance

To further investigate the role of the high baseline levels of Atg3 seen in ERPC9 cells, ERPC9 cells were transfected with Atg3 siRNA and treated appropriately. siRNA transfection was successful in blocking Atg3 translation and autophagy activity (represented by low LC3II and high p62 levels in relative cells without Atg3 siRNA) as seen in Figure 7A. There was no significant difference in cell survival in control group of ERPC9 cells transfected with non-specific siRNA (NS siRNA) compared to ERPC9 cells without transfection (p = 0.445). However, with Atg3 siRNA transfection, ERPC9 cells were re-sensitized to erlotinib-alone treatment and had an associated significant increase in cell death of 32.0% compared to erlotinib-alone treated ERPC9 cells with NS siRNA transfection (p = 0.043). ERPC9 cells also became significantly more sensitive to combination treatment after Atg3 siRNA transfection when compared to NS siRNA transfected cells; 10.3% more cell death was observed (p = 0.002) (Figure 7B). However, this phenomenon was not seen in cisplatin treated ERPC9 cells transfected with Atg3 siRNA; there was no significant difference between cisplatin treated Atg3 siRNA and NS siRNA (70.2% vs. 70.8%, respectively) (p = 0.924). This data suggests and supports that the baseline up-regulation of Atg3 is intimately involved in ER and cell survival and blocking the translation of Atg3 in ERPC9 cells is able to reverse ER and confer re-sensitivity to erlotinib treatments. In addition, blocking Atg3 did not confer increase sensitive to cisplatin drug treatment, which suggests that inhibition of Atg3 is specific for ER.

Atg3 siRNA transfection was able to produce a significant increase in sensitivity towards single treatments in the parental PC9 cell line. Erlotinib with Atg3 siRNA was able to induce 11.5% more cell death compared to NS siRNA erlotinib treatment (p<0.0001) and cisplatin with Atg3 siRNA was able induce 9.7% more cell death compared to NS siRNA cisplatin treatment (p<0.0001). The difference in cell death between combination treated cells with Atg3 siRNA compared to combination treatment with NS siRNA was small (4.5%); although this difference was significant (p = 0.002).

### Apoptosis

Apoptosis was measured through western blot analysis of cleaved caspase 3 and cleaved PARP. There were significantly lower levels of cleaved PARP (p = 0.004) and cleaved caspase 3 (p<0.0001) in ERPC9 cells compared to PC9 cells (Figure 8B and 8C, respectively). When ERPC9 cells were treated with erlotinib-cisplatin combination treatment, significantly higher levels of cleaved PARP and cleaved caspase 3 were observed compared to ERPC9 cells treated with control (p<0.0001, and p<0.0001, respectively), erlotinib-alone (p = 0.033, and p = 0.001, respectively), and cisplatin-alone (p = 0.004 and p<0.0001, respectively) (Figure 8A and 8D). This suggests that erlotinib-cisplatin combination treatment induces cell death through the promotion of apoptosis.

## Discussion

In this study, we demonstrated that low dose erlotinib-cisplatin combination is able to overcome ER and induce synergistic cell death in both ERPC9 and the parental PC9 cell line while remaining relatively non-toxic to NHBE cells. We also identified an increased baseline level of autophagy that may act as a protective mechanism underlying ER in EGFR mutated lung adenocarcinoma. The modulation of autophagy consequently altered the cell’s sensitivity to erlotinib: the induction of autophagy led to greater resistance and the inhibition of autophagy led to less resistance (more sensitivity to erlotinib). Upon further examination, a higher baseline level of Atg3 was revealed and specific inhibition of Atg3 resulted in the reversal of ER and re-sensitization to erlotinib treatment. It also seems that Atg3 inhibition may be specific to erlotinib as blocking Atg3 in cells treated with cisplatin had no additional killing effects. This result supports that up-regulation of Atg3 may be the key to conferring higher basal levels of autophagy leading to ER. It also seems that erlotinib-cisplatin combination treatment works by targeting the high basal autophagy level, specifically Atg3. These results lead us to deduce that Atg3 may be a key regulator/promoter of ER in EGFR overexpressed lung adenocarcinoma and targeting baseline over expression/translation of Atg3 may be able to result in the reversal of ER.

There have been few studies that use combinations of chemotherapy and anticancer agents in the treatment of TKI resistant NSCLC. According to those studies available in the current literature, it seems that the overall effectiveness of combination treatment depends on the cancer’s profile and specific drugs used. Okabe *et al*. demonstrated that combination gefitinib and 5-flurouracil (5FU) was able to produce synergistic cell death in cell lines that had acquired MET-amplification, but not in other cell lines [33] and Kim *et al.* showed combination gefitinib and cetuximab was not able to produce synergistic cell death, but lapatinib with cetuximab did [34]. In addition to providing additional killing properties, combination erlotinib and paclitaxel was shown to be effective in delaying TKI resistance [35].

Similarly, our findings suggest that combination regimens may be effective against TKI resistant lung cancers. More specifically, the combination of erlotinib and cisplatin was able to induce synergistic cell death in erlotinib resistant lung adenocarcinoma (ERPC9 cells). Interestingly, the observed synergistic effect was greater in ERPC9 cells (CI = 0.12) than in PC9 cells (CI = 0.45) further indicating that this regimen may be in fact effective in erlotinib resistant lung adenocarcinoma. While it remains to be determined how combination treatment is able to target Atg3-medaited autophagy to overcome ER, the synergy of combination treatment may be due to how each drug targets separate pathways that modulate a similar endpoint, DNA damage and repair. In the setting of environmental exposures to toxic agents that are able to induce DNA damage such as heat, radiation, hydrogen peroxide, and chemotherapeutic agents most notably cisplatin, cancer cells are able to activate a nuclear EGFR pathway in which promotes DNA repair and cell survival [36]. However, in the setting of exposure to EGFR inhibitors, such as erlotinib, the nuclear EGFR pathway is inhibited and DNA repair mechanisms through this pathway are abated. This eventually could lead to a greater susceptibility to cisplatin DNA damage and cancer cell death in addition to what erlotinib does alone. Furthermore, the effectiveness of erlotinib-cisplatin combination may be explained by its ability to not only induced toxic cell death, but also apoptotic cell death as demonstrated by our findings.

Just as importantly, low dose combination treatment was not toxic to non-cancer cells. In fact, in a phase I and II clinical trial for the treatment of metastatic head and neck squamous cell carcinoma, the combination of erlotinib and cisplatin was reported to have a favorable toxicity profile [37], supporting our *in vitro* findings. Taken together, our results suggest low dose erlotinib-cisplatin combination therapy may be an effective regimen in overcoming ER in EGFR mutated lung adenocarcinoma, be effective in treatment of sensitive cancers, and at the same time have minimal toxicity and side effects even when using both agents.

Identifying and understanding the mechanisms of cancer resistance are critical in order to design effective therapies to overcome such resistance. Currently reported and identified mechanisms of drug resistance in lung cancer, not specific to TKI, include increased efflux of drugs due to overly expressed drug transporters, alterations in cell cycle regulatory proteins and/or checkpoints, defects in apoptosis signaling pathways such as anti-apoptotic protein bcl-2 overexpression, and over expression of DNA repair mechanisms (such as nucleotide excision-repair, mismatch-repair, base excision-repair, non-homologous end-joining, and homologous-recombination) [38]–[45]. More specific to TKI resistance, the two most accepted and recognized mechanisms in EGFR mutated NSCLC includes T790M point mutation in the EGFR kinase domain (which accounts about approximately 50%) and MET oncogene amplification (accounting about 20%) [46], [47]. The other relatively less established mechanism consist of AXL tyrosine kinase receptor overexpression, altered EGFR trafficking, overexpression of insulin-like growth factor-1 (IGF1), amplification of mutated EGFR downstream signaling pathways, and expression of ABCG2 efflux of drug transporters [48]–[52]. However, to our knowledge, no study has reported the involvement of autophagy in ER for NSCLC.

The “dual role” of autophagy in cancer continues to be a controversial topic. Several studies have shown that autophagy may work as a protective mechanism for cancer cells in stressful and/or harmful environment such as exposure to chemotherapies and even act as a possible mechanism of chemotherapy resistance [23], [53], [54]. However, others have reported that autophagy may work as a tumor suppressor mechanism and promote cell death by working through Beclin-1 [55], [56], considered as a type II programmed cell death pathway [20], [57], [58], and prevent cancer cell proliferation through cell cycle arrest and therefore causing senescence [58], [59]. Autophagy modulation, mainly induction, by various drug treatment in NSCLC cell lines have been demonstrated such as with cetuximab in HCC827 cells [22], MG-2477 (tubulin inhibitor) in A549 cells [60], and PM02734 (Elisidepsin) in A549 nude mice [61]. However, the relationship of combination therapies and autophagy has not been studied previously. Contrary to the studies mentioned, our study showed decreased levels in autophagy after ERPC9 cells received erlotinib-cisplatin combination treatment. In addition, the findings in this study suggests that autophagy acts as an essential mechanism underlying ER as demonstrated by increased cell survival when treated with an autophagy promoting agent (rapamycin), and decreased cell survival when autophagy was inhibited with 3-MA.

While in this study 3-MA-alone and combination treatment-alone are individually able to cause a little over 30% cell death in ERPC9 cells (as seen in Figure 4B), the combination of 3-MA and erlotinib-cisplatin treatment effect was greater but less than additive. A possible explanation for this is correlated with our finding that combination treatment targets Atg3. 3-MA and combination treatment are mechanistically acting on the same autophagy pathway, but at different levels in the pathway. While 3-MA inhibits PI3K class III (which is upstream of Atg3), its observed effectiveness will be minimalized unless its effect is much greater than the combination effects down-stream of Atg3 from the combination treatment. Effectively, the agents are blocking the same pathway twice; hence, the full effects of each treatment individually are not seen. The 30% cell death seen with 3-MA was somewhat expected as other studies have shown that 3-MA is able to induce cell death in cancer cells as well [62]. More specifically, the blockage of autophagosome formation with 3-MA in anti-estrogen resistant breast cancer cells blocked growth and induced significant cell death [63]. Furthermore, several in vivo studies have shown high levels of autophagosome formation in viable tumor cells [64] further supporting that autophagy may be a crucial mechanism in maintaining viability of cancer cells.

The current literature has not examined or reported which protein(s) in the autophagy pathway are able to confer and/or contribute to chemotherapy resistance. There have been brief descriptions of the functions of the various Atg proteins. Atg7 is described as an E-1 like enzyme essential in autophagosome formation [65], axon membrane turnover, and maintenance of hematopoietic stem cells [66], [67]. The Atg5-12 conjugate is involved in the conversion of LC3I to LC3II [68] and modulation of the innate immune system in responses to antivirus [69]. Atg3 is less understood, and currently the only recognized function of Atg3 is to convert LC3I to LC3II in the promotion of autophagy [32]. In this study, we showed higher levels of Atg3 in ERPC9 cells compared to PC9 cells and the inhibition of Atg3 translation resulted in re-sensitized to erlotinib-alone treatment in ERPC9 cells. Atg3 most likely did not demonstrate an additional function given the associated expected changes in LC3I and LC3II and the presence of high baseline level of Atg3. The cause of the high Atg3 level is unclear at this time and may involve over-expression of the Atg3 gene or over-translation, consequently leading to higher autophagy activity. It is also important to determine whether the significantly higher levels of Atg3-mediated autophagy is secondarily due to a specific mechanism of acquired resistance (additional genetic alteration leading to advantageous mutations) or a general mechanism of adaptive resistance that arises *de novo* in cancer cells. With further planned elucidation of how Atg3 is up-regulated, this will allow us to answer this question in the future.

We acknowledge that this study is focused on a single erlotinib-resistant cell line and therefore may not be generalizable to all erlotinib-resistant lung cancer patients. It is important to note that there has not been an identified mechanism of resistance that is generalizable to all cancer cell lines or types. Rather, more frequent mechanisms of resistance exist and we expect that autophagy as a mechanism of resistance is not the exception. Given that we tested a single cell line it remains unclear whether autophagy is common to many cell lines or strictly a mechanism of resistance seen in EGFR mutated lung adenocarcinoma and it is unlikely that all cancer cell lines harbor autophagy as a sole mechanism of resistance. Despite this, up to 80% of adenocarcinoma (not limited to lung cancer) harbor EGFR mutations and it is possible that autophagy as a mechanism of acquired resistance is also present in other EGFR mutated cancers. In addition, given that we also show autophagy may be involved not only with ER but also cancer cell survival in general, it is probable that many other cancers harbor a similar mechanism of resistance. Further studies of resistance in other lung adenocarcinoma cell lines and cancer cell types are needed to characterize the frequency in which autophagy acts as a mechanism of resistance. Nonetheless the hope of this study is to introduce autophagy as a new mechanism of TKI resistance in non-small cell lung cancer and further pilot additional studies of autophagy’s role in resistance not only in lung cancer, but other cancer types as well.

In this study, we revealed a novel role for Atg3 in the promotion of ER as the inhibition of Atg3 translation was able to result in the re-sensitization of erlotinib resistant cells to erlotinib-alone treatment. Also, we demonstrated that erlotinib-cisplatin combination is an effective treatment against erlotinib resistant cancer cells by targeting autophagy through down-regulation of Atg3 and induction of apoptotic cell death. Continued research on how Atg3 is up-regulated and how autophagy promotes cancer resistances are needed. Future pharmaceutical development and clinical management may utilize this new finding in order to develop novel treatments against NSCLC and ER.
